# Systematic Review and Meta-Analysis of the Benefit and Safety of Preoperative Administration of Steroid in Patients Undergoing Liver Resection

**DOI:** 10.3389/fphar.2019.01442

**Published:** 2019-11-28

**Authors:** Lingpeng Yang, Zifei Zhang, Junjie Kong, Wentao Wang

**Affiliations:** ^1^Department of Liver Surgery, Liver Transplantation Center, West China Hospital of Sichuan University, Chengdu, China; ^2^Department of General Surgery, The Affiliated Hospital of Xizang Minzu University, Xianyang, China

**Keywords:** liver resection, steroid, inflammatory response, complications, meta-analysis, systematic review

## Abstract

**Objective:** To evaluate the benefit and safety of preoperative administration of steroid in patients undergoing liver resection.

**Methods:** Randomized controlled trials (RCTs) which comparing preoperative administration of steroid in patients undergoing liver resection with control group were identified through a systematic literature search in PubMed, Embase, and Cochrane Library Central databases. This meta-analysis was carried out to assess the liver function, inflammatory response, and postoperative complications after liver surgery.

**Results:** Six RCTs including 411 patients were reviewed. The pooled result showed that there was no significant difference in the incidence of overall complications between the steroid group and the control group (OR, 0.57; 95% CI, 0.27–1.17; *P* = 0.13). With respect to specific complications, no significant difference was detected between the two groups in infection complications (OR, 0.95; 95% CI, 0.13–6.95; *P* = 0.96), wound complications (OR, 0.65; 95% CI, 0.32–1.33; *P* = 0.24), liver failure (OR, 0.41; 95% CI, 0.10–1.64; *P* = 0.21), bile leakage (OR, 0.57; 95% CI, 0.17–1.89; *P* = 0.36), and pleural effusion (OR, 1.24; 95% CI, 0.55–2.78; *P* = 0.60). For liver function, the level of serum total bilirubin (TB) on postoperative day 1 (POD 1) was significantly decreased associated with the intervention of steroid (MD, −0.54; 95% CI, −0.94 to −0.15; *P* = 0.007). However, no significant difference was found in the level of alanine aminotransferase (ALT) (MD, −69.39; 95% CI, −226.52 to 87.75; *P* = 0.39) and aspartate aminotransferase (AST) (MD, −93.44; 95% CI, −275.68 to 88.80; *P* = 0.31) on POD 1 between the two groups. Serum IL-6 level on POD 1 (MD, −57.98; 95% CI, −73.04 to −42.91; *P* < 0.00001) and CRP level on POD 3 (MD, −4.83; 95% CI, −6.07 to −3.59; *P* < 0.00001) were significantly reduced in the steroid group comparing to the control group. Compared with the control group, the level of early postoperative IL-10 was significant higher in the steroid group (MD, 17.89; 95% CI, 3.89 to 31.89; *P* = 0.01).

**Conclusion:** Preoperative administration of steroid in liver resection can promote the recovery of liver function and inhibit the inflammatory response without increasing postoperative complications. Further studies should focus on determining which patients would benefit most from the steroid.

## Introduction

Major abdominal surgery such as liver resection results in an acute systemic inflammatory response characterized by hemodynamic and metabolic changes that produce and release various chemical mediators, including stress hormones, free radicals, and cytokines (Faist et al., 1996; Kohl and Deutschman, 2006). During liver resection, in order to reduce intraoperative blood loss, blockage to the inflow blood of the liver is needed (Pringle maneuver). The downside of this technique is the ischemia-reperfusion (IR) damage to liver cells (Teoh and Farrell, 2003). Inflammatory cytokines play a significant role in this process. Increased levels of inflammatory cytokines are connected with high postoperative mortality and morbidity (Baigrie et al., 1992). Consequently, more attention has evolved to regulating inflammatory responses that probably harmful to the surgery. Many protective measures have been applied to reduce hepatic IR injury, including intermittent or selective Pringle maneuver (Figueras et al., 2005; Kim et al., 2007) and multiple pharmacological interventions such as erythropoietin, vitamin E, branched chain amino acids, prostaglandin E (Bartels et al., 2004; Kawano et al., 2005; Ishikawa et al., 2010; Kato et al., 2010).

The properties of anti-inflammatory and immune modulating allow steroid to be widely used in a variety of diseases associated with inflammatory response (Barnes, 1998; Sapolsky et al., 2000). Some previous studies have showed that preoperative use of steroid may reduce pro-inflammatory cytokines, alleviate liver IR injury, maintain a stable coagulation function (Pulitano et al., 2007a; Saidi et al., 2007). However, steroid is a double-edged sword also with lots of drawbacks, which may lead to delayed wound healing, hyperglycemia, postoperative infection, impaired liver regeneration, and the reactivation of hepatitis virus (Holte and Kehlet, 2002; Polderman et al., 2019). The potential side effects of preoperative steroid use are of great concern in liver surgery. Considering the advantages and disadvantages, the use of steroid before liver resection is still controversial owing to absence of standard treatment guideline. Although some meta-analyses have discussed this topic (Li et al., 2013; Richardson et al., 2014), the authors ignored the high heterogeneity in the pooled results. Besides, the authors only analyzed the overall complications and they did not analyze the specific complications which were the significant parameters to evaluate the safety of preoperative steroid use. It is necessary to perform an updated and comprehensive meta-analysis by including the latest studies. Therefore, we performed this meta-analysis to evaluate the benefit and safety of preoperative administration of steroid in patients undergoing liver resection and provide a reference for clinical practice.

## Materials And Methods

### Literature Search Strategy

This systematic review and meta-analysis was performed in accordance with the Preferred Reporting Items for Systematic Reviews and Meta-Analyses (PRISMA) Statement (Liberati et al., 2009). A systematic literature search was conducted independently by two authors (LY and ZZ) in the PubMed, Embase, and Cochrane Library Central databases through 4 May 2019. The search strategies were based on combinations of the following keywords: “hepatectomy” OR “liver resection” OR “hepatic resection” OR “liver surgery” OR “surgery of the liver” AND “steroids” OR “cortisone” OR “corticosteroids” OR “glucocorticoid” OR “glucocorticosteroids” OR “methylprednis” OR “methylprednisolone” OR “predniso” OR “prednisone” OR “dexamethasone” OR “hydrocortisone” In addition, a manual search of all references of retrieved articles was performed. The literature search was restricted to human studies and articles published in English.

### Inclusion and Exclusion Criteria

Only randomized controlled trials (RCTs) comparing preoperative use of steroid in patients undergoing liver resection with no use of steroid were included in this review. Exclusion criteria were: (1) retrospective studies, case-control studies and cohort studies, (2) liver transplantation studies, and (3) studies that did not directly investigate IR injury.

### Data Extraction

The following data were extracted independently by the same two authors, and disagreements were resolved by a third author (WW). Extracted data were: (1) study characteristics: first author, country, study design, publication year, and indications for liver resection; (2) patient characteristics: age, sex, number of patients in steroid group and control group, number of patients in each group divided according to Child-Pugh classification, and number of patients in each group divided based on underlying liver disease; (3) intraoperative data: operative time, hepatic ischemia time, intraoperative blood loss, method of vascular control, liver resection technique, number of major resection in each group; (4) postoperative data: total bilirubin (TB), C-reactive protein (CRP), interleukin (IL) 6, IL-10, alanine aminotransferase (ALT), aspartate aminotransferase (AST), postoperative hospital stay, and postoperative complications (overall complications, infection complications, wound complications, liver failure, bile leakage, and pleural effusion). Whenever relevant data were required, the corresponding authors were contacted *via* email. If contact was failed, data were measured from enlarged figures.

### Statistical Analysis

All statistical analyses were performed using Review Manager (Version 5.3, Cochrane Collaboration, Oxford, England). The odds ratio (OR) and the mean difference (MD) with 95% confidence interval (CI) were used for dichotomous data and continuous data, respectively. Statistical analysis was performed with data mean and standard deviation for continuous data. If included studies provided only medians and data ranges, the means ± standard deviation were calculated using the methods described by Hozo et al. (2005). Statistical heterogeneity was assessed by I^2^ value, an I^2^ value greater than 50% was regarded as significant heterogeneity (Higgins and Thompson, 2002). A random-effects model was selected in the case of significant heterogeneity. A *P* value < 0.05 was considered to be statistically significant. The results were illustrated by forest plots. The quality of included studies was evaluated using Cochrane Collaboration’s Risk of Bias Tool (Higgins et al., 2011). Subgroup analyses were performed to seek potential heterogeneity source and identify subsets of patients who tended to benefit from steroid according to region (Japan, Italy, or Germany), method of vascular control (no Pringle maneuver, continuous or intermittent Pringle maneuver), sample size (>40 or <40), and drug regimen (dosage of 30 mg/kg or dosage of 500 mg). Sensitivity analyses were also performed by omitting the included studies in turn to analyze the stability of the pooled results.

## Results

### Study Selection and Characteristics

**Figure 1:**
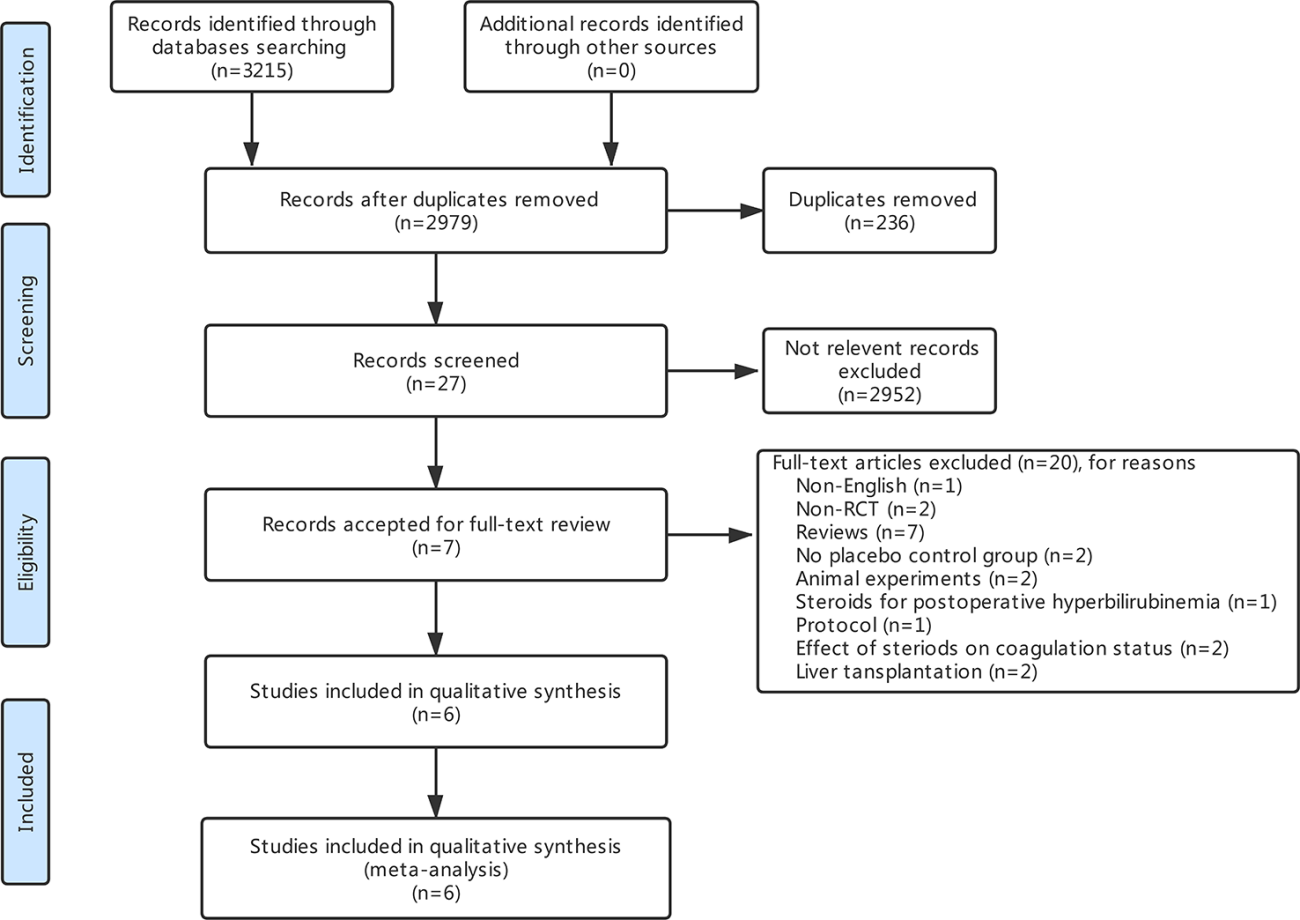
Flow diagram of the study retrieval and selection process.

The search strategy identified 3,215 articles, of which 236 were duplicates, 2,952 were not relevant to the subject and 20 did not fulfill the inclusion criteria. Total seven articles considered for inclusion (Yamashita et al., 2001; Muratore et al., 2003; Aldrighetti et al., 2006; Pulitano et al., 2007b; Schmidt et al., 2007; Hayashi et al., 2011; Donadon et al., 2016). However, two studies (Aldrighetti et al., 2006; Pulitano et al., 2007b) came from the same institution, including a part of overlapping patients, more detailed study was included (Aldrighetti et al., 2006). Finally, six studies meet the inclusion criteria was included in this meta-analysis (Yamashita et al., 2001; Muratore et al., 2003; Aldrighetti et al., 2006; Schmidt et al., 2007; Hayashi et al., 2011; Donadon et al., 2016). Study selection process is shown in **Figure 1** following PRISMA guidelines (Liberati et al., 2009). The publication time of these studies ranged from 2001 to 2016. Of the six included studies, three came from Italy, two from Japan, and one from Germany. A total 411 patients were included in this meta-analysis, of whom 206 were in the steroid group and 205 in the control group. Main characteristics of the included studies are summarized in **Table 1**. Characteristics of liver resection in the included studies are shown in **Table 2**.

**Table 1:** Main characteristics of the included studies.

Author	Year	Country	Study design	No. of patients		Age (year)		Gender, M/F		Child-Pugh classification (A/B/C)		Steroid protocol	Outcome measure
				Steroid	Control	Steroid	Control	Steroid	Control				
Aldrighetti et al.	2006	Italy	RCT	36	37	61.8 (21–78)^a^	63 (31–85)^a^	22/14	23/14	36/0/0	37/0/0	MP 500 mg before anesthesia	ALT, AST, TB, INR, IL-6, complications, hospital stay
Donadon et al.	2016	Italy	RCT	16	16	65 (27–80)^a^	63 (22–77)^a^	10/6	9/7	NR	NR	MP 500 mg 1h before liver resection	ALT, TB, complications, hospital stay
Hayashi et al.	2011	Japan	RCT	102	98	69 (39–81)^a^	70 (35–72)^a^	NR	NR	99/3/0	94/4/0	HC 500 mg before hepatic pedicle clamping, 300 mg on POD 1, 200 mg on POD 2, 100 mg on POD 3	ALT, AST, TB, INR, CRP, IL-6, IL-10, complications, hospital stay
Muratore et al.	2003	Italy	RCT	25	28	65.4 ± 10.8^b^	64.1 ± 11.7^b^	17/8	11/17	NR	NR	MP 30 mg/kg 0.5 h before liver resection	ALT, AST, TB, PT, IL-6, complications, hospital stay
Schmidt et al.	2007	Germany	RCT	10	10	57^c^	65^c^	3/7	4/6	NR	NR	MP 30 mg/kg 1.5 h before surgery	ALT, AST, TB, INR, IL-6, IL-8, IL-10, TNF-α, CRP, complications, hospital stay
Yamashita et al.	2001	Japan	RCT	17	16	60.3 ± 1.8^b^	56.8 ± 3.9^b^	13/4	11/5	NR	NR	MP 500 mg 2 h before surgery	ALT, AST, TB, PT, IL-6, IL-10, CRP, complications, hospital stay

^a^Data expressed as median (range); ^b^data are expressed as mean ± SD; ^c^data are expressed as mean.

RCT, randomized controlled trial; MP, methylprednisolone; HC, hydrocortisone; ALT, alanine aminotransferase; AST, aspartate aminotransferase; INR, international normalized ratio; CRP, C-reactive protein; IL, interleukin; TNF, tumor necrosis factor; PT, prothrombin time; NR, not reported.

**Table 2:** Characteristics of liver resection of the included studies.

Author	Year	Major resection		Vascular control	Resection technique	Indication of surgery (HCC/MLT/CCC/LDLT/GC/others)		Operative time (min)		Ischemia time (min) Steroid Control		Blood loss (mL)		Underlying liver disease (normal/cirrhosis/steatosis)	
		Steroid	Control			Steroid	Control	Steroid	Control	Steroid	Control	Steroid	Control	Steroid	Control
Aldrighetti et al.	2006	26	27	Intermittent Pringle maneuver	Ultrasonic dissector and ultrasonic scalpel	14/14/4/0/0/4	12/16/4/0/1/4	408 (240–460)^a^	440 (220–480)^a^	52.4 (20–89)^a^	43 (20–78)^a^	621 (350–720)^a^	662 (300–800)^a^	18/14/4	21/12/4
Donadon et al.	2016	7	5	Intermittent Pringle maneuver	Crush clamping	6/6/4/0/0/0	2/12/0/0/0/2	383.5(235–546)^a^	351 (226–640)^a^	83 (46–162)^a^	80.5 (35–168)^a^	275 (100–1000)^a^	200 (0–700)^a^	NR	NR
Hayashi et al.	2011	11	15	Intermittent Pringle maneuver	NR	63/32/6/0/0/1	66/23/5/0/0/4	330 (165–834)^a^	316 (136–697)^a^	72 (0–247)^a^	60 (0–203)^a^	324 (5–1577)^a^	257 (10–1972)^a^	NR	NR
Muratore et al.	2003	13	15	Continuous Pringle maneuver	Ultrasonic dissector	NR	NR	NR	NR	41.4 ± 15.9^b^	37.3 ± 17.8^b^	322.8 ± 261.4^b^	294.6 ± 271.9^b^	8/10/7	15/4/9
Schmidt et al.	2007	6	5	No use	Ultrasound dissector	2/4/0/0/0/4	1/4/2/0/0/3	222^c^	252^c^	NR	NR	340^c^	780^c^	NR	NR
Yamashita et al.	2001	5	6	Continuous Pringle maneuver	NR	13/0/0/4/0/0	8/3/1/4/0/0	338 ± 87^b^	352 ± 56^b^	NR	NR	892 ± 437^b^	822 ± 220^b^	NR	NR

^a^Data expressed as median (range); ^b^data are expressed as mean ± SD; ^c^data are expressed as mean.

HCC, hepatocellular carcinoma; MLT, metastatic liver tumor; CCC, cholangiocellular carcinoma; LDLT, living donor liver transplantation; GC, gallbladder carcinoma; NR, not reported.

### Postoperative Complications

**Figure 2:**
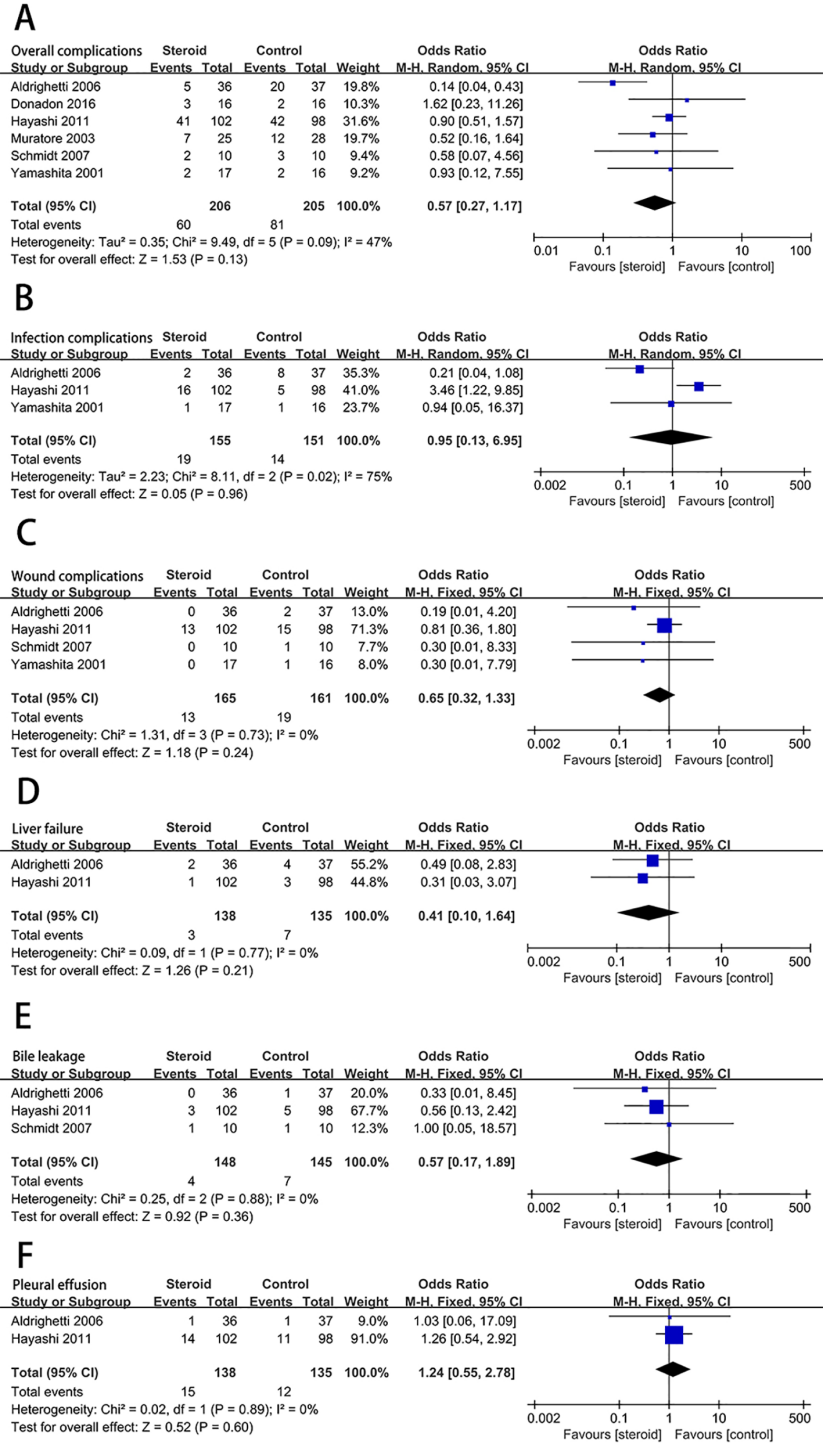
The pooled results of comparison between the steroid group and the control group for postoperative complications illustrated by forest plots. The results were overall complications **(A)**, infection complications **(B)**, wound complications **(C)**, liver failure **(D)**, bile leakage **(E)**, pleural effusion **(F)**.

Postoperative complications reported in the included studies are summarized in **Table 3**. There was no significant difference in the incidence of overall complications between the steroid group and the control group (OR, 0.57; 95% CI, 0.27–1.17; *P* = 0.13) (**Figure 2A**). In terms of specific complications, no significant difference was detected between the two groups in infection complications (OR, 0.95; 95% CI, 0.13–6.95; *P* = 0.96) (**Figure 2B**), wound complications (OR, 0.65; 95% CI, 0.32–1.33; *P* = 0.24) (**Figure 2C**), liver failure (OR, 0.41; 95% CI, 0.10–1.64; *P* = 0.21) (**Figure 2D**), bile leakage (OR, 0.57; 95% CI, 0.17–1.89; *P* = 0.36) (**Figure 2E**), and pleural effusion (OR, 1.24; 95% CI, 0.55–2.78; *P* = 0.60) (**Figure 2F**). There were no postoperative deaths in all studies.

**Table 3:** Postoperative complications reported in the included studies.

Complications (n)	Aldrighetti et al. (2006)		Donadon et al. (2016)		Hayashi et al. (2011)		Muratore et al. (2003)		Schmidt et al. (2007)		Yamashita et al. (2001)	
	SG	CG	SG	CG	SG	CG	SG	CG	SG	CG	SG	CG
Infection complications	2	8	NR	NR	16	5	NR	NR	—	—	1	1
Liver failure	2	4	NR	NR	1	3	NR	NR	—	—	—	—
Bile leakage	0	1	NR	NR	3	5	NR	NR	1	1	—	—
Ascites	—	—	NR	NR	—	—	NR	NR	—	—	1	0
Wound complications	0	2	NR	NR	13	15	NR	NR	0	1	0	1
Hemorrhage	0	1	NR	NR	—	—	NR	NR	—	—	—	—
Cardiovascular	0	3	NR	NR	—	—	NR	NR	—	—	—	—
Pleural effusion	1	1	NR	NR	14	11	NR	NR	—	—	—	—
Bile duct stenosis	—	—	NR	NR	—	—	NR	NR	1	1	—	—
Reoperation	—	—	NR	NR	1	3	NR	NR	—	—	—	—
Atelectasis	—	—	NR	NR	5	8	NR	NR	—	—	—	—
Side effect of steroids	—	—	NR	NR	—	—	NR	NR	—	—	—	—
Death	—	—	NR	NR	—	—	NR	NR	—	—	—	—
Total events	5	20	3	2	53	50	7	12	2	3	2	2
Overall complications*	5	20	3	2	41	42	7	12	2	3	2	2

NR, not reported; SG, steroid group; CG, control group.

*Number of patients.

### Postoperative Liver Function

**Figure 3:**
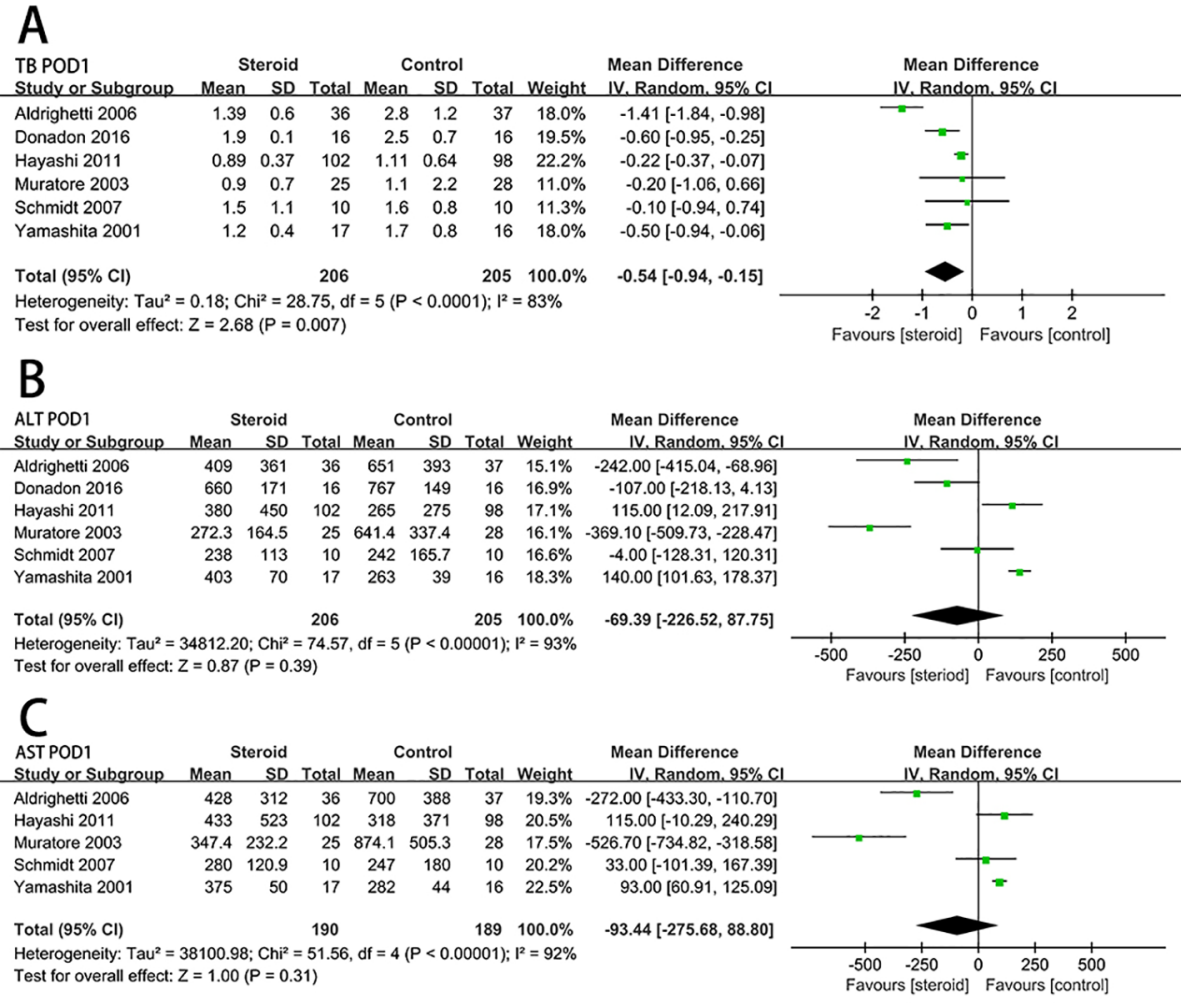
The pooled results of comparison between the steroid group and the control group for postoperative liver function illustrated by forest plots. The results were TB on POD 1 **(A)**, ALT on POD 1 **(B)**, AST on POD 1 **(C)**.

The level of serum TB on postoperative day 1 (POD 1) was significantly decreased associated with the intervention of steroids (MD, −0.54; 95% CI, −0.94 to −0.15; *P* = 0.007) (**Figure 3A**). There was no significant difference in the level of ALT (MD, −69.39; 95% CI, −226.52 to 87.75; *P* = 0.39) (**Figure 3B**) and AST (MD, −93.44; 95% CI, −275.68 to 88.80; *P* = 0.31) (**Figure 3C**) on POD 1 between the steroid group and the control group.

### Postoperative Inflammatory Response

**Figure 4:**
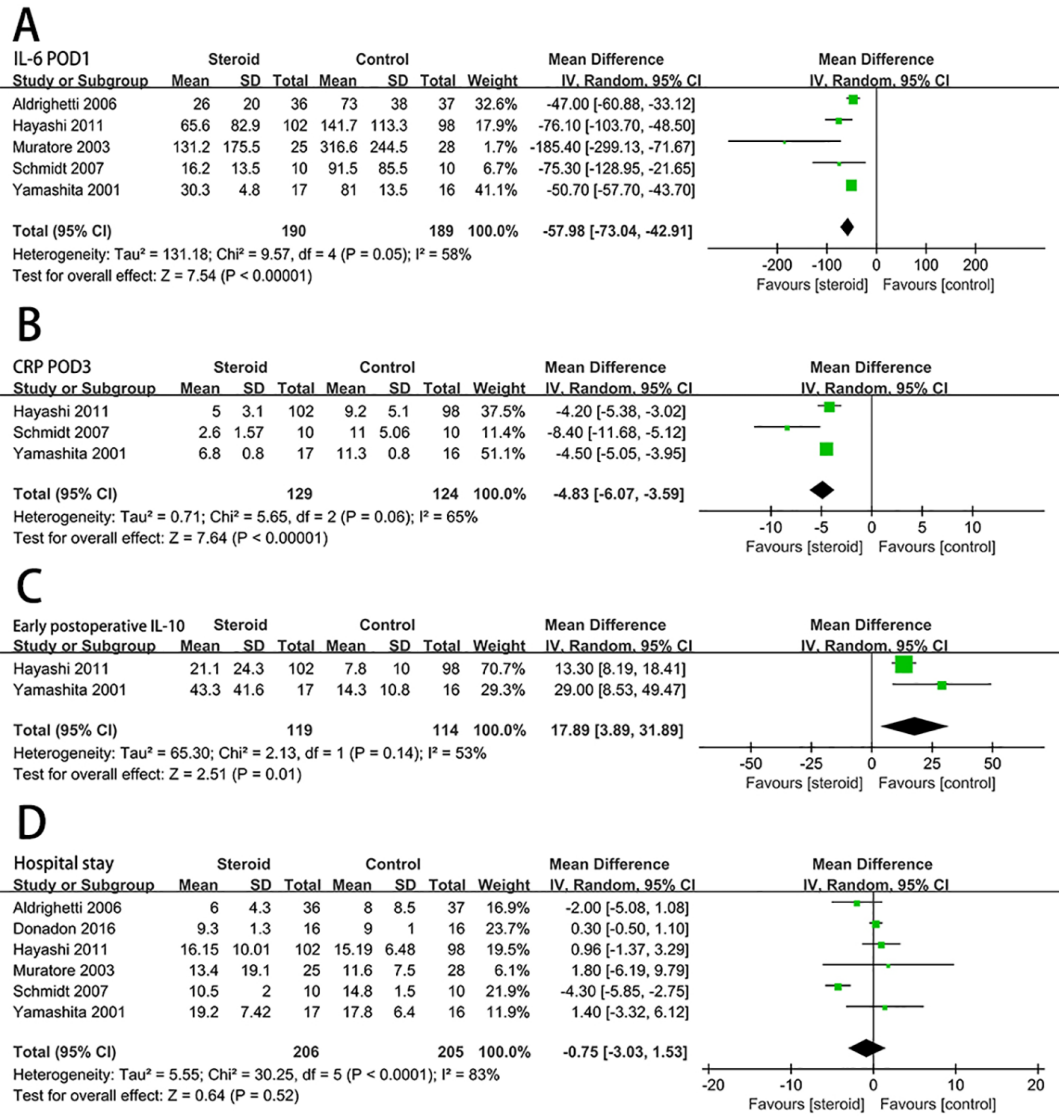
The pooled results of comparison between the steroid group and the control group for postoperative inflammatory response and length of hospital stay illustrated by forest plots. The results were IL-6 on POD 1 **(A)**, CRP on POD 3 **(B)**, early postoperative IL-10 **(C)**, length of hospital stay **(D)**.

Serum IL-6 level on POD 1 (MD, −57.98; 95% CI, −73.04 to −42.91; *P* < 0.00001) (**Figure 4A**) and CRP level on POD 3 (MD, −4.83; 95% CI, −6.07 to −3.59; *P* < 0.00001) (**Figure 4B**) were significantly reduced in the steroid group comparing to the control group. Compared with the control group, the level of early postoperative IL-10 was significant higher in the steroid group (MD, 17.89; 95% CI, 3.89 to 31.89; *P* = 0.01) (**Figure 4C**).

### Hospital Stay

For length of hospital stay, there was no statistically significant difference between the steroid group and the control group (MD, −0.75; 95% CI, −3.03 to 1.53; *P* = 0.52) (**Figure 4D**).

### Quality of the Included Studies

**Figure 5:**
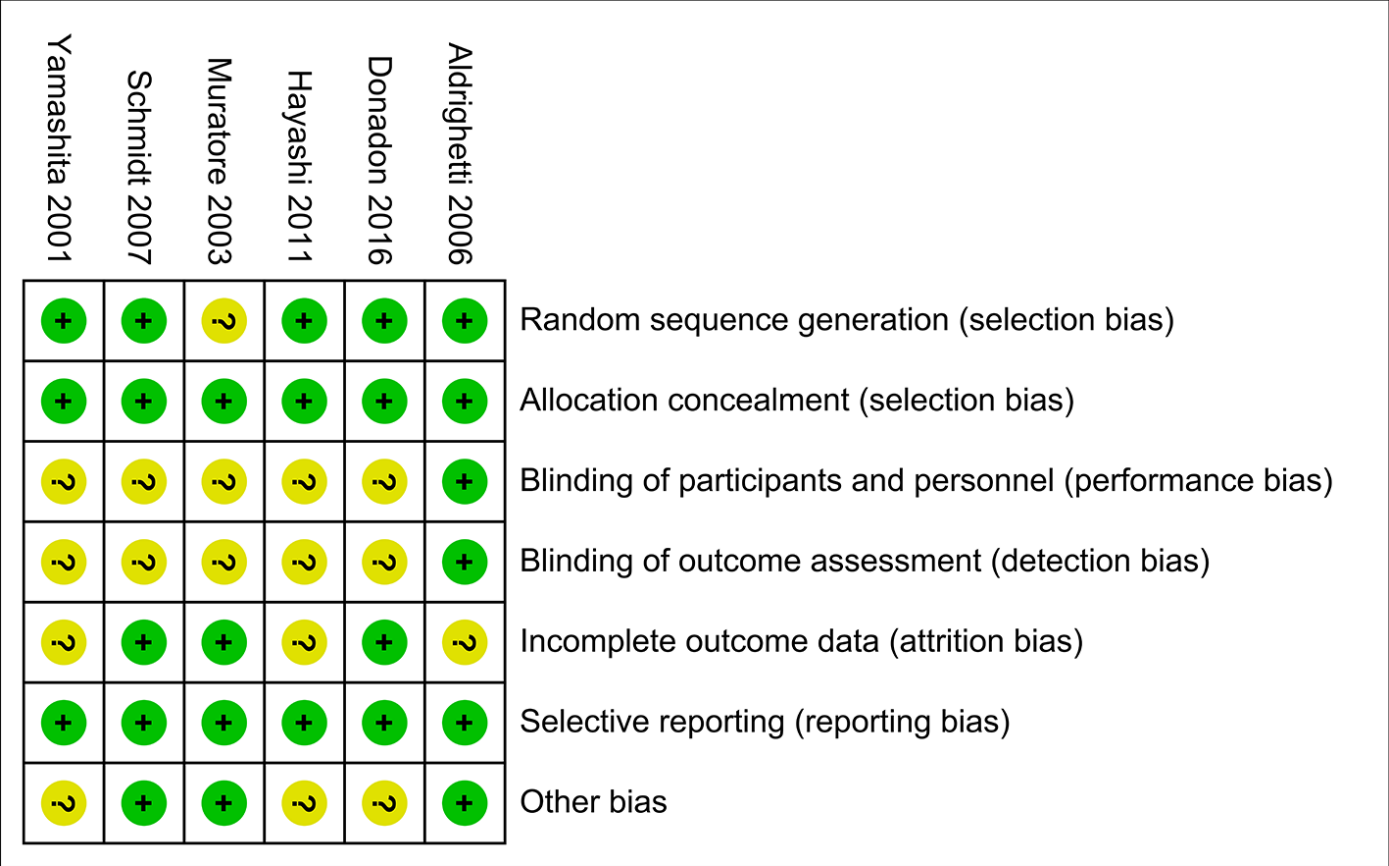
Risk of bias summary. Dash sign: high risk of bias; plus sign: low risk of bias; question mark sign: unclear risk of bias.

The evaluation of the risk of bias for included studies is shown in **Figure 5** according to the Cochrane Collaboration’s Risk of Bias Tool. Due to a small number of included studies, the test power was insufficient and the symmetry was difficult to evaluate, funnel plot analysis was not carried out.

### Subgroup Analysis

**Figure 6:**
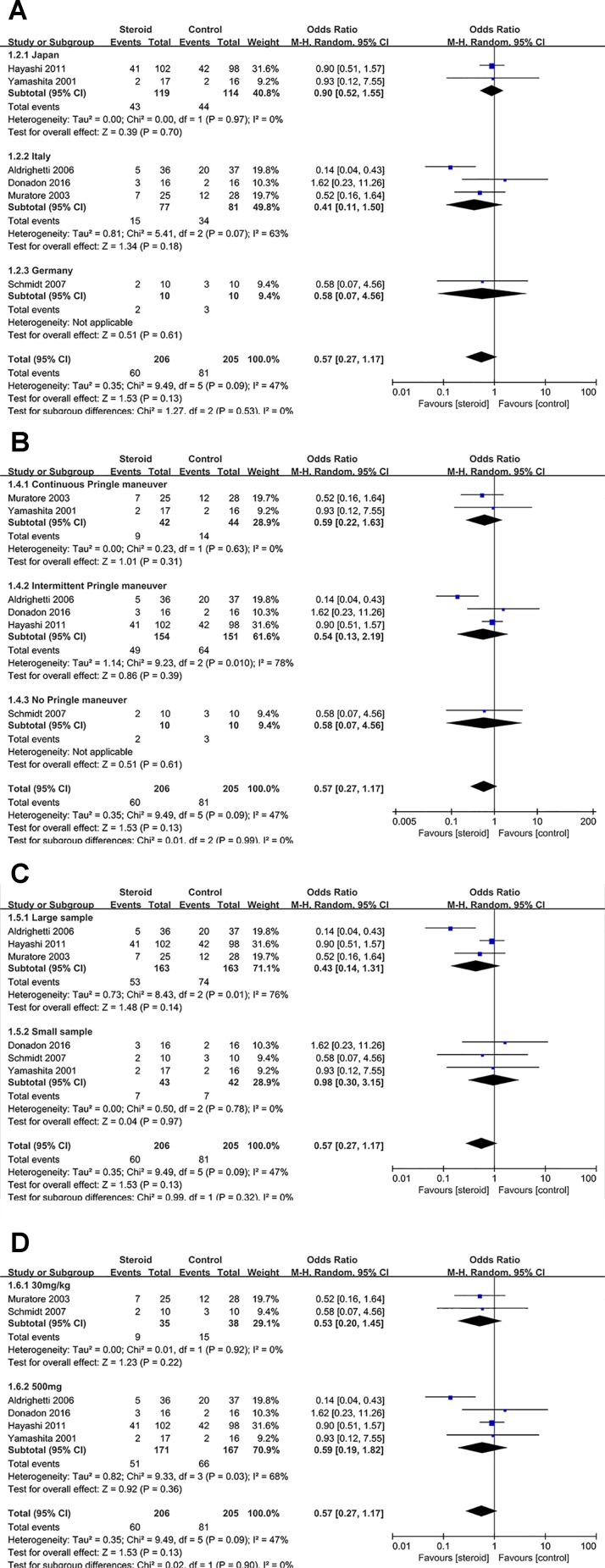
Subgroup analysis of overall complications stratified by region **(A)**, method of vascular control **(B)**, sample size **(C)**, and drug regimen **(D)**.

**Figure 7:**
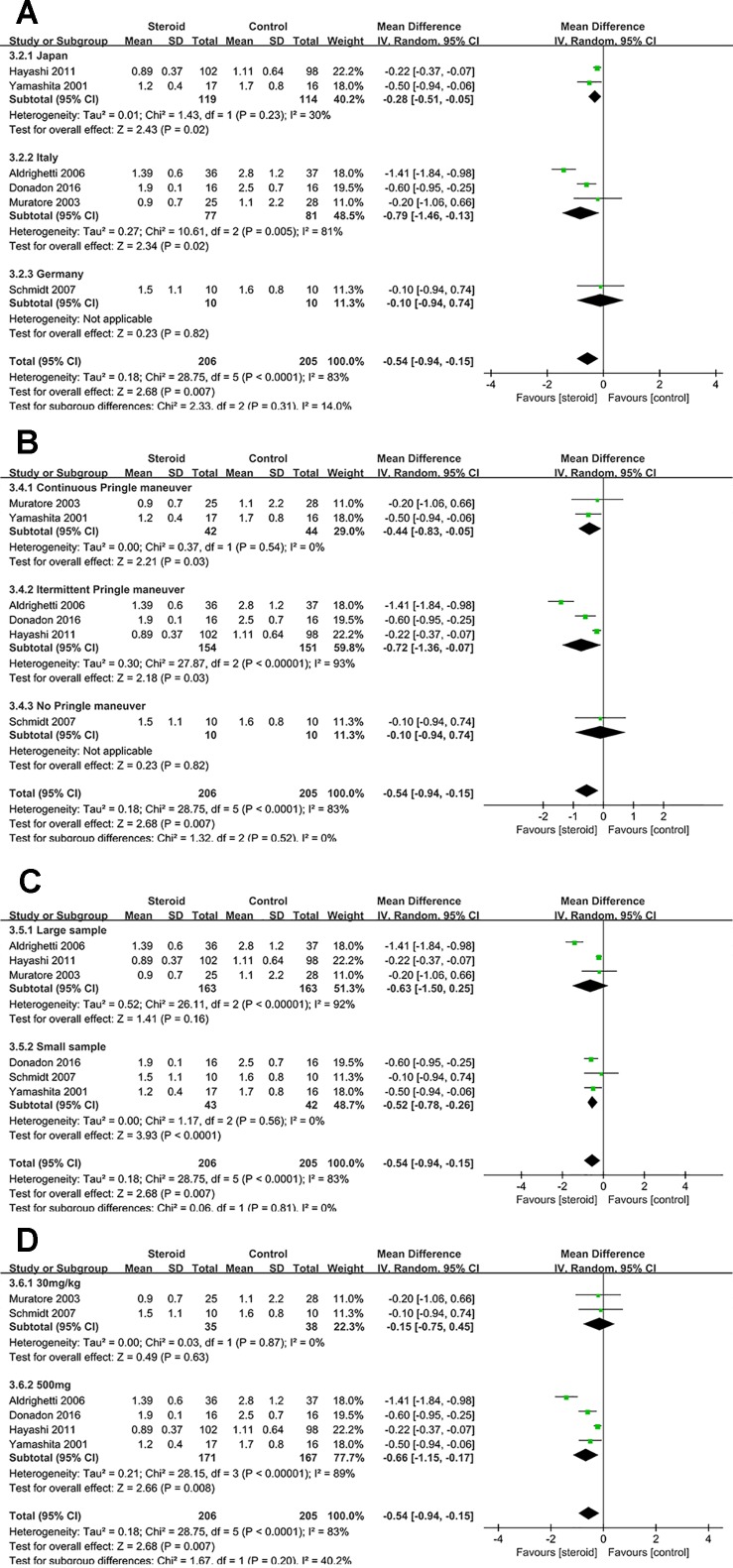
Subgroup analysis of TB on POD 1 stratified by region **(A)**, method of vascular control **(B)**, sample size **(C)**, and drug regimen **(D)**.

**Figure 8:**
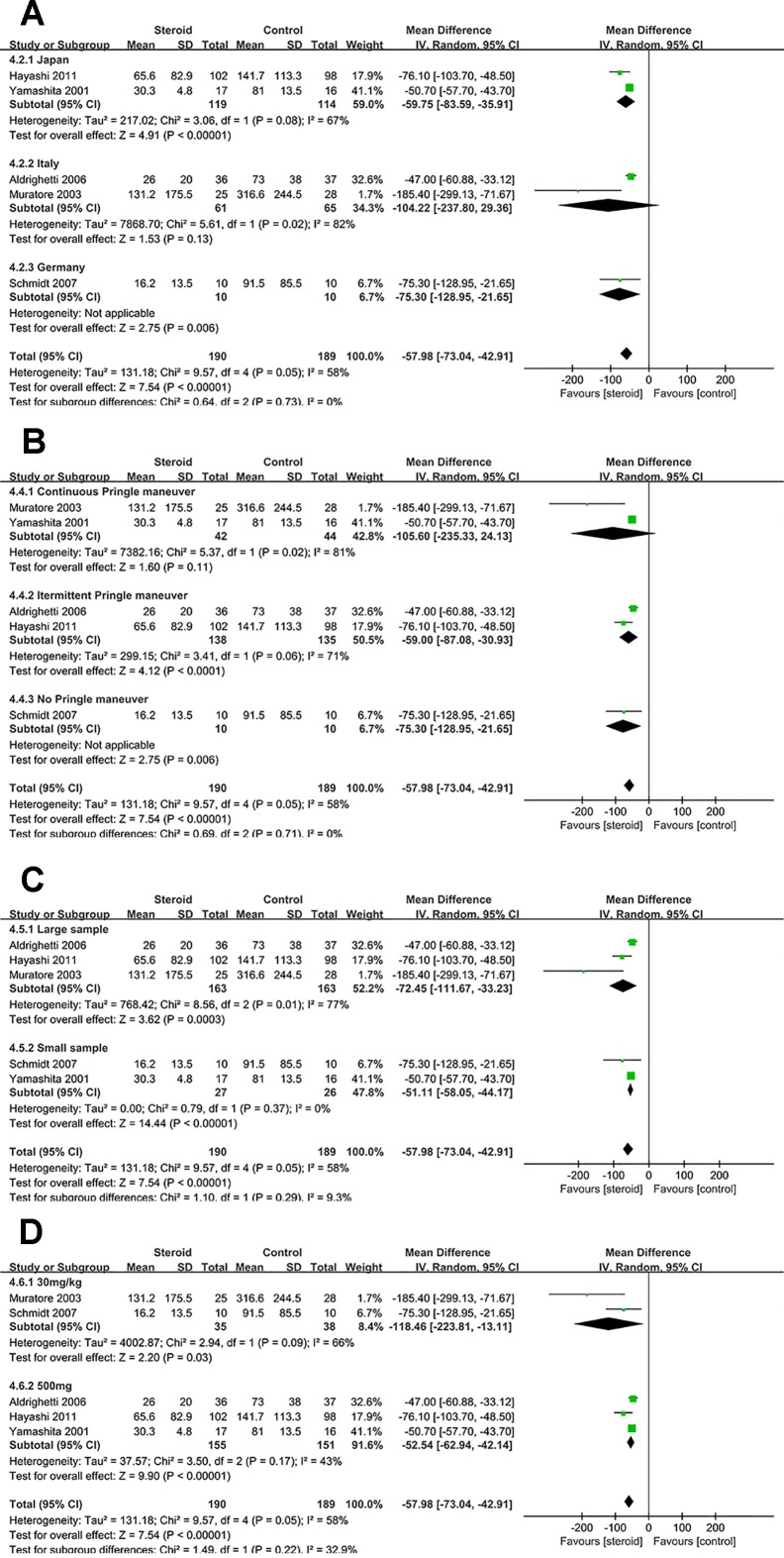
Subgroup analysis of IL-6 on POD 1 stratified by region **(A)**, method of vascular control **(B)**, sample size **(C)**, and drug regimen **(D)**.

Subgroup analyses were conducted for overall complications (**Figure 6**), TB on POD 1 (**Figure 7**), and IL-6 on POD 1 (**Figure 8**) based on region, method of vascular control, sample size, and drug regimen. The summary of subgroup analyses are presented in **Table 4**.

**Table 4:** Summary of the subgroup analysis.

Outcome	Subgroup	No. of studies	MD/OR (95% CI)	P value for overall effect	I^2^ value	P value for heterogeneity
Overall complications	Studies from Japan	2	0.90 (0.52, 1.55)	0.70	0	0.97
	Studies from Italy	3	0.41 (0.11, 1.50)	0.18	63%	0.07
	Studies from Germany	1	0.58 (0.07, 4.56)	0.61	—	—
	Studies with continuous Pringle maneuver	2	0.59 (0.22, 1.63)	0.31	0	0.63
	Studies with intermittent Pringle maneuver	3	0.54 (0.13, 2.19)	0.39	78%	0.010
	Studies without Pringle maneuver	1	0.58 (0.07, 4.56)	0.61	—	—
	Studies with large sample (>40)	3	0.43 (0.14, 1.31)	0.14	76%	0.01
	Studies with small sample (<40)	3	0.98 (0.30, 3.15)	0.97	0	0.78
	Studies with dosage of 30 mg/kg	2	0.53 (0.20, 1.45)	0.22	0	0.92
	Studies with dosage of 500 mg	4	0.59 (0.19, 1.82)	0.36	68%	0.03
TB on POD 1	Studies from Japan	2	−0.28 (−0.51, −0.05)	**0.02**	30%	0.23
	Studies from Italy	3	−0.79 (−1.46, −0.13)	**0.02**	81%	0.005
	Studies from Germany	1	−0.10 (−0.94, 0.74)	0.82	—	—
	Studies with continuous Pringle maneuver	2	−0.44 (−0.83, −0.05)	**0.03**	0	0.54
	Studies with intermittent Pringle maneuver	3	−0.72 (−1.36, −0.07)	**0.03**	93%	*P* < 0.00001
	Studies without Pringle maneuver	1	−0.10 (−0.94, 0.74)	0.82	—	—
	Studies with large sample (>40)	3	−0.63 (−1.50, 0.25)	0.16	92%	*P* < 0.00001
	Studies with small sample (<40)	3	−0.52 (−0.78, −0.26)	***P* < 0.0001**	0	0.56
	Studies with dosage of 30 mg/kg	2	−0.15 (−0.75, 0.45)	0.63	0	0.87
	Studies with dosage of 500 mg	4	−0.66 (−1.15, −0.17)	**0.008**	89%	*P* < 0.00001
IL-6 on POD 1	Studies from Japan	2	−59.75 (−83.59, −35.91)	***P* < 0.00001**	67%	0.08
	Studies from Italy	2	−104.22 (−237.80, 29.36)	0.13	82%	0.02
	Studies from Germany	1	−75.30 (−128.95, −21.65)	**0.006**	—	—
	Studies with continuous Pringle maneuver	2	−105.60 (−235.33, 24.13)	0.11	81%	0.02
	Studies with intermittent Pringle maneuver	2	−59.00 (−87.08, −30.93)	***P* < 0.0001**	71%	0.06
	Studies without Pringle maneuver	1	−75.30 (−128.95, −21.65)	**0.006**	—	—
	Studies with large sample (>40)	3	−72.45 (−111.67, −33.23)	**0.0003**	77%	0.01
	Studies with small sample (<40)	2	−51.11 (−58.05, −44.17)	***P* < 0.00001**	0	0.37
	Studies with dosage of 30 mg/kg	2	−118.46 (−223.81, −13.11)	**0.03**	66%	0.09
	Studies with dosage of 500 mg	3	−52.54 (−62.94, −42.14)	***P* < 0.00001**	43%	0.17

Bold text indicates statistical significance; RCT, randomized controlled trial; MD, mean difference; OR, odds ratio; CI, confidence interval.

### Subgroup Analysis of Overall Complications

No significant change was detected in the subgroup analysis of overall complications, the results were consistent with the aforementioned outcome. However, low heterogeneity of overall complications was found in studies from Japan (*I* ^2^ = 0), studies with continuous Pringle maneuver (*I* ^2^ = 0), studies with small sample (*I* ^2^ = 0), and studies with dosage of 30 mg/kg (*I* ^2^ = 0), indicating that region, method of vascular control, sample size, and drug regimen were potential sources of heterogeneity.

### Subgroup Analysis of TB on POD 1

The difference of TB on POD 1 was not significant between the steroid group and the control group in studies with large sample or studies with dosage of 30 mg/kg. Low heterogeneity of TB on POD 1 was detected in studies with continuous Pringle maneuver (*I* ^2^ = 0), studies with small sample (*I* ^2^ = 0), and studies with dosage of 30 mg/kg (*I* ^2^ = 0), implying that method of vascular control, sample size, and drug regimen were potential sources of heterogeneity.

### Subgroup Analysis of IL-6 on POD 1

The difference of IL-6 on POD 1 was not significant between the two groups in studies from Italy or studies with continuous Pringle maneuver. Low heterogeneity of IL-6 on POD 1 was observed in studies with small sample (*I* ^2^ = 0), suggesting that sample size was potential source of heterogeneity.

### Sensitivity Analysis

Sensitivity analyses were also performed for overall complications, TB on POD 1, and IL-6 on POD 1, in which one study was removed at a time to assess the stability of the pooled results. Sensitivity analyses showed that pooled results did not change significantly with exclusion of the included studies in turn. The pooled results of overall complications, TB on POD 1, and IL-6 on POD 1 ranged from (OR, 0.46; 95% CI, 0.19–1.12; *I* ^2^ = 37%; *P* = 0.09), (MD, −0.64; 95% CI, −1.08 to −0.19; *I* ^2^ = 73%; *P* = 0.005), (MD, −71.11; 95% CI, −102.48 to −39.75; *I* ^2^ = 67%; *P* < 0.00001) to (OR, 0.83; 95% CI, 0.52–1.32; *I* ^2^ = 0; *P* = 0.43), (MD, −0.34; 95% CI, −0.52 to −0.15; *I* ^2^ = 21%; *P* = 0.0004), (MD, −53.03; 95% CI, −62.65 to −43.41; *I* ^2^ = 30%; *P* < 0.00001).

## Discussion

In the present study, we performed a meta-analysis of preoperative administration of steroid in liver resection, demonstrating that steroid can promote the recovery of liver function and inhibit the inflammatory response without increasing postoperative complications after hepatectomy.

Serum TB is an important clinical parameter for postoperative liver dysfunction and also a critical prognosis relevant factor. The level of TB on POD 1 was significantly decreased associated with the use of steroid. The lower TB level in steroid group might be the result of a faster recovery of the liver. However, for the level of ALT and AST on POD 1, no statistical difference was detected between the steroid group and the control group according to the pooled results. This may be caused by different composition of patients in each included study. The study by Muratore et al. ( 2003) indicates that in patients with chronic liver diseases and AST and ALT levels on POD 1 were significantly lower in the steroid group than the control group. Nevertheless, in patients with normal liver, no statistical difference was found. Hence, the patients with chronic liver diseases may benefit more from preoperative administration of steroid and the steroid should be more often implemented in these patients.

As one of the most vital organs, the liver plays an important role in metabolic, secretory, and endocrine, which producing inflammatory factors, modifying coagulation balance, and regulating protein metabolism (Berger et al., 1997). Cytokines released from stimulated macrophages and monocytes after liver resection, this reaction is called the hepatic acute response. In the acute response, IL-6 acts as a critical modulator of the inflammation, not only influencing B-lymphocytes and T-lymphocytes, but inducing the production of acute-phase proteins in the liver, such as CRP, antiproteinases, and fibrinogen (Menger and Vollmar, 2004). Elevated blood cytokines can increase the risk of postoperative complications (Kimura et al., 2006), therefore treatments to limit the cytokine response is needed. With the administration of steroid, IL-6, and CRP were significantly suppressed in the steroid group comparing to the control group. Previous research showed that the release of IL-6 is associated with the extent of liver resection, duration of operation, and volume of blood loss (Biffl et al., 1996). And the two included studies (Aldrighetti et al., 2006; Donadon et al., 2016) demonstrated that protective effects of preoperative steroid administration were more conspicuous in patients with larger resection volume and longer ischemic time. In liver resection, the release of IL-6 is augmented by the use of Pringle maneuver, owing to the IR injury. Both continuous and intermittent Pringle maneuver were used in the included studies. However, the subgroup analysis of IL-6 on POD 1 demonstrated that steroid can lead to a better outcome in studies with intermittent Pringle maneuver. IL-10 is a potent anti-inflammatory cytokine produced primarily by activated macrophages after liver surgery, it inhibits the release of pro-inflammatory cytokines and therefore tends to down-regulate the inflammatory reaction (Jerin et al., 2003). In this meta-analysis, use of the steroid was relevant to a significant increase of serum IL-10 level in early postoperative period. Consequently, maintaining the balance of pro- and anti-inflammatory cytokines is a key factor to the recovery of patients undergoing liver surgery.

Prophylactic administration of steroid may potentially induce unnecessary side effects, such as postoperative infection, delayed wound healing, impaired liver regeneration. However, our pooled results indicated that preoperative use of steroid have no effect on the incidence of overall complications between the steroid group and the control group. In terms of specific complications, steroid also does not increase the incidence of infection complications, wound complications, liver failure, bile leakage, and pleural effusion. Besides, the subgroup analyses of overall complications suggested similar results. One (Aldrighetti et al., 2006) of the included studies reported that the infective complications were more frequent in the control group than the steroid group, yet other studies demonstrated no statistical difference was found between the two groups. This result was seemly paradoxical, however, Yamashita et al. (Yamashita et al., 2001) previously reported that preoperative use of steroid can decrease the serum level of immunosuppressive antigen and the positive rate of serum candida antigen, a marker of bacterial translocation. And similar findings were reported in esophagectomy (Shimada et al., 2000). Takeda et al. (Takeda et al., 2002) previously proposed the non regeneration type liver failure was mainly induced by severe IR injury after hepatectomy based on histological findings. And some literature reported that the overproduction of IL-6 during IR injury may inhibit liver regeneration (Wustefeld et al., 2000). Steroid can suppress IL-6 production, therefore, preoperative administration of steroid may have a positive effect on liver failure after liver resection. Though this meta-analysis showed that no significant difference was detected in the incidence of liver failure between the two groups. Despite great technical advances, bile leakage after liver resection remains a main postoperative complication. A previous study reported that immunosuppression may repress cholangiocyte regeneration by inhibiting signal transducer and activator of transcription 3 activation in a rat liver transplantation model (Chen et al., 2010). So far, no research show any available evidence about the effect of steroids on cholangiocyte regeneration. The pooled result of our meta-analysis indicated that there was no significant difference in bile leakage between the two groups.

Long-term follow-up is required for these patients after liver resection, because there are some concerns about the risk of tumor recurrence may cause by the anti-inflammatory effect of steroid. Recently, Ogasawara et al. (2017) reported that prophylactic administration of steroid in patients undergoing transcatheter arterial chemoembolization can achieve a greater overall complete response rate than control group. This result indicated that a short-term administration of steroid did not significantly affect tumor treatment. But more research is needed to solve this issue.

Some limitations existed in this meta-analysis. First, most of the studies included in this meta-analysis were relatively small sample sizes and lack of long-term follow-up data. Large sample size RCTs with long-term follow-up are urgently needed in the future. Second, postoperative complications were vital parameters to evaluate the safety of preoperative steroid administration, but only four studies reported specific complications. Third, some date were extracted from graphs in original studies, because we failed to contact with corresponding author. This method may cause measurement bias.

In conclusion, preoperative administration of steroid in liver resection can promote the recovery of liver function and inhibit the inflammatory response without increasing postoperative complications. The steroid effect was associated with underlying liver disease and liver surgery itself (extent of liver resection and ischemic time), whether the steroid should be used routinely in all patients before liver resection remains unresolved. Further studies are required to explore this strategy.

## Author Contributions

WW and LY designed the study. LY and ZZ identiﬁed studies and extracted data, disagreements were resolved by discussion with WW. JK and ZZ performed the statistical analyses and prepared the relevant tables and graphs, LY drafted the manuscript and WW revised it. All authors read and approved the ﬁnal version of the manuscript

## Funding

This study was funded by the National Natural Science Foundation of China (No. 81770566).

## Conflict of Interest

The authors declare that the research was conducted in the absence of any commercial or financial relationships that could be construed as a potential conflict of interest.

## Abbreviations

IR, ischemia reperfusion; RCT, randomized controlled trial; TB, total bilirubin; CRP, C-reactive protein; IL-6, interleukin 6; IL-10, interleukin 10; ALT, alanine aminotransferase; AST, aspartate aminotransferase; OR, odds ratio; MD, mean difference; CI, confidence interval; POD, postoperative day.

## References

[B1] Aldrighetti L. Pulitano C. Arru M. Finazzi R. Catena M. Soldini L. (2006). Impact of preoperative steroids administration on ischemia-reperfusion injury and systemic responses in liver surgery: a prospective randomized study. Liver Transpl. 12, 941–949. 10.1002/lt.20745 16710858

[B2] Baigrie R. J. Lamont P. M. Kwiatkowski D. Dallman M. J. Morris P. J. (1992). Systemic cytokine response after major surgery. Br. J. Surg. 79, 757–760. 10.1002/bjs.1800790813 1393463

[B3] Barnes P. J. (1998). Anti-inflammatory actions of glucocorticoids: molecular mechanisms. Clin. Sci. (Lond) 94, 557–572. 10.1042/cs0940557 9854452

[B4] Bartels M. Biesalski H. K. Engelhart K. Sendlhofer G. Rehak P. Nagel E. (2004). Pilot study on the effect of parenteral vitamin E on ischemia and reperfusion induced liver injury: a double blind, randomized, placebo-controlled trial. Clin. Nutr. 23, 1360–1370. 10.1016/j.clnu.2004.05.003 15556258

[B5] Berger D. Bolke E. Seidelmann M. Beger H. G. (1997). Time-scale of interleukin-6, myeloid related proteins (MRP), C reactive protein (CRP), and endotoxin plasma levels during the postoperative acute phase reaction. Shock 7, 422–426. 10.1097/00024382-199706000-00006 9185242

[B6] Biffl W. L. Moore E. E. Moore F. A. Peterson V. M. (1996). Interleukin-6 in the injured patient. Marker of injury or mediator of inflammation? Ann. Surg. 224, 647–664. 10.1097/00000658-199611000-00009 8916880 PMC1235442

[B7] Chen L. P. Zhang Q. H. Chen G. Qian Y. Y. Shi B. Y. Dong J. H. (2010). Rapamycin inhibits cholangiocyte regeneration by blocking interleukin-6-induced activation of signal transducer and activator of transcription 3 after liver transplantation. Liver Transpl. 16, 204–214. 10.1002/lt.21985 20104495

[B8] Donadon M. Molinari A. F. Corazzi F. Rocchi L. Zito P. Cimino M. (2016). Pharmacological modulation of ischemic-reperfusion injury during pringle maneuver in hepatic surgery. a prospective randomized pilot study. World J. Surg. 40, 2202–2212. 10.1007/s00268-016-3506-1 27094558

[B9] Faist E. Schinkel C. Zimmer S. (1996). Update on the mechanisms of immune suppression of injury and immune modulation. World J. Surg. 20, 454–459. 10.1007/s002689900071 8662134

[B10] Figueras J. Llado L. Ruiz D. Ramos E. Busquets J. Rafecas A. (2005). Complete versus selective portal triad clamping for minor liver resections: a prospective randomized trial. Ann. Surg. 241, 582–590. 10.1097/01.sla.0000157168.26021.b8 15798459 PMC1357061

[B11] Hayashi Y. Takayama T. Yamazaki S. Moriguchi M. Ohkubo T. Nakayama H. (2011). Validation of perioperative steroids administration in liver resection: a randomized controlled trial. Ann. Surg. 253, 50–55. 10.1097/SLA.0b013e318204b6bb 21233606

[B12] Higgins J. P. Thompson S. G. (2002). Quantifying heterogeneity in a meta-analysis. Stat. Med. 21, 1539–1558. 10.1002/sim.1186 12111919

[B13] Higgins J. P. Altman D. G. Gotzsche P. C. Juni P. Moher D. Oxman A. D. (2011). The Cochrane Collaboration’s tool for assessing risk of bias in randomised trials. BMJ 343, d5928. 10.1136/bmj.d5928 22008217 PMC3196245

[B14] Holte K. Kehlet H. (2002). Perioperative single-dose glucocorticoid administration: pathophysiologic effects and clinical implications. J. Am. Coll. Surg. 195, 694–712. 10.1016/s1072-7515(02)01491-6 12437261

[B15] Hozo S. P. Djulbegovic B. Hozo I. (2005). Estimating the mean and variance from the median, range, and the size of a sample. BMC Med. Res. Methodol. 5, 13. 10.1186/1471-2288-5-13 15840177 PMC1097734

[B16] Ishikawa Y. Yoshida H. Mamada Y. Taniai N. Matsumoto S. Bando K. (2010). Prospective randomized controlled study of short-term perioperative oral nutrition with branched chain amino acids in patients undergoing liver surgery. Hepatogastroenterology 57, 583–590. 20698232

[B17] Jerin A. Pozar-Lukanovic N. Sojar V. Stanisavljevic D. Paver-Erzen V. Osredkar J. (2003). Balance of pro- and anti-inflammatory cytokines in liver surgery. Clin. Chem. Lab. Med. 41, 899–903. 10.1515/CCLM.2003.136 12940515

[B18] Kato M. Sawada T. Kita J. Shimoda M. Kubota K. (2010). Erythropoietin ameliorates early ischemia-reperfusion injury following the Pringle maneuver. World J. Gastroenterol. 16, 4838–4845. 10.3748/wjg.v16.i38.4838 20939113 PMC2955254

[B19] Kawano T. Hosokawa N. Maruta T. Maruta N. Takasaki M. (2005). [Reevaluation of protective effects of alprostadil on hepatic function in patients undergoing hepatectomy]. Masui 54, 982–991. 16167789

[B20] Kim Y. I. Fujita S. Hwang Y. J. Chun J. M. Song K. E. Chun B. Y. (2007). Successful intermittent application of the Pringle maneuver for 30 minutes during human hepatectomy: a clinical randomized study with use of a protease inhibitor. Hepatogastroenterology 54, 2055–2060. 18251159

[B21] Kimura F. Shimizu H. Yoshidome H. Ohtsuka M. Kato A. Yoshitomi H. (2006). Circulating cytokines, chemokines, and stress hormones are increased in patients with organ dysfunction following liver resection. J. Surg. Res. 133, 102–112. 10.1016/j.jss.2005.10.025 16386757

[B22] Kohl B. A. Deutschman C. S. (2006). The inflammatory response to surgery and trauma. Curr. Opin. Crit. Care 12, 325–332. 10.1097/01.ccx.0000235210.85073.fc 16810043

[B23] Li H. Wei Y. Li B. (2013). Preoperative steroid administration in liver resection: a systematic review and meta-analysis. Hepatogastroenterology 60, 160–169. 10.5754/hge12498 22829554

[B24] Liberati A. Altman D. G. Tetzlaff J. Mulrow C. Gotzsche P. C. Ioannidis J. P. (2009). The PRISMA statement for reporting systematic reviews and meta-analyses of studies that evaluate health care interventions: explanation and elaboration. Ann. Intern. Med. 151, W65–W94. 10.7326/0003-4819-151-4-200908180-00136 19622512

[B25] Menger M. D. Vollmar B. (2004). Surgical trauma: hyperinflammation versus immunosuppression? Langenbecks Arch. Surg. 389, 475–484. 10.1007/s00423-004-0472-0 15173946

[B26] Muratore A. Ribero D. Ferrero A. Bergero R. Capussotti L. (2003). Prospective randomized study of steroids in the prevention of ischaemic injury during hepatic resection with pedicle clamping. Br. J. Surg. 90, 17–22. 10.1002/bjs.4055 12520569

[B27] Ogasawara S. Chiba T. Ooka Y. Kanogawa N. Motoyama T. Suzuki E. (2017). A randomized placebo-controlled trial of prophylactic dexamethasone for transcatheter arterial chemoembolization. Hepatology 67, 575–585. 10.1002/hep.29403 28746788

[B28] Polderman J. Farhang-Razi V. van Dieren S. Kranke P. DeVries J. H. Hollmann M. W. (2019). Adverse side-effects of dexamethasone in surgical patients—an abridged Cochrane systematic review. Anaesthesia. 74, 929–939. 10.1111/anae.14610 30821852

[B29] Pulitano C. Aldrighetti L. Arru M. Finazzi R. Catena M. Guzzetti E. (2007a). Preoperative methylprednisolone administration maintains coagulation homeostasis in patients undergoing liver resection: importance of inflammatory cytokine modulation. Shock 28, 401–405. 10.1097/shk.0b013e318063ed11 17577134

[B30] Pulitano C. Aldrighetti L. Arru M. Finazzi R. Soldini L. Catena M. (2007b). Prospective randomized study of the benefits of preoperative corticosteroid administration on hepatic ischemia-reperfusion injury and cytokine response in patients undergoing hepatic resection. HPB (Oxford) 9, 183–189. 10.1080/13651820701216984 18333219 PMC2063598

[B31] Richardson A. J. Laurence J. M. Lam V. W. (2014). Use of pre-operative steroids in liver resection: a systematic review and meta-analysis. HPB (Oxford) 16, 12–19. 10.1111/hpb.12066 23461716 PMC3892310

[B32] Saidi R. F. Chang J. Verb S. Brooks S. Nalbantoglu I. Adsay V. (2007). The effect of methylprednisolone on warm ischemia-reperfusion injury in the liver. Am. J. Surg. 193, 345–347; discussion 347-8. 10.1016/j.amjsurg.2006.09.017 17320532

[B33] Sapolsky R. M. Romero L. M. Munck A. U. (2000). How do glucocorticoids influence stress responses? Integrating permissive, suppressive, stimulatory, and preparative actions. Endocr. Rev. 21, 55–89. 10.1210/edrv.21.1.0389 10696570

[B34] Schmidt S. C. Hamann S. Langrehr J. M. Hoflich C. Mittler J. Jacob D. (2007). Preoperative high-dose steroid administration attenuates the surgical stress response following liver resection: results of a prospective randomized study. J. Hepatobiliary Pancreat. Surg. 14, 484–492. 10.1007/s00534-006-1200-7 17909718

[B35] Shimada H. Ochiai T. Okazumi S. Matsubara H. Nabeya Y. Miyazawa Y. (2000). Clinical benefits of steroid therapy on surgical stress in patients with esophageal cancer. Surgery 128, 791–798. 10.1067/msy.2000.108614 11056442

[B36] Takeda K. Togo S. Kunihiro O. Fujii Y. Kurosawa H. Tanaka K. (2002). Clinicohistological features of liver failure after excessive hepatectomy. Hepatogastroenterology 49, 354–358. 11995449

[B37] Teoh N. C. Farrell G. C. (2003). Hepatic ischemia reperfusion injury: pathogenic mechanisms and basis for hepatoprotection. J. Gastroenterol. Hepatol. 18, 891–902. 10.1046/j.1440-1746.2003.03056.x 12859717

[B38] Wustefeld T. Rakemann T. Kubicka S. Manns M. P. Trautwein C. (2000). Hyperstimulation with interleukin 6 inhibits cell cycle progression after hepatectomy in mice. Hepatology 32, 514–522. 10.1053/jhep.2000.16604 10960443

[B39] Yamashita Y. Shimada M. Hamatsu T. Rikimaru T. Tanaka S. Shirabe K. (2001). Effects of preoperative steroid administration on surgical stress in hepatic resection: prospective randomized trial. Arch. Surg. 136, 328–333. 10.1001/archsurg.136.3.328 11231856

